# NT-proBNP and cardiac cycle efficiency changes during extubation process in critically ill patients

**DOI:** 10.1186/cc12099

**Published:** 2013-03-19

**Authors:** F Franchi, P Mongelli, M Cozzolino, B Galgani, C Ragozzino, C Bianchi, B Biagioli

**Affiliations:** 1 University of Siena, Italy

## Introduction

Several factors can lead to weaning failure from mechanical ventilation (MV). Among these, cardiac dysfunction is one of the main causes. NT-proBNP has been proposed as a biomarker of cardiovascular function during weaning from MV. Unfortunately it does not provide for a continuous monitoring of cardiac function. Pulse wave analysis may serve as a continuous bedside monitoring tool of cardiovascular performance. Cardiac cycle efficiency (CCE) is an indirect index of left ventricular performance obtained by the pulse contour method MostCare (Vygon, Padova, Italy). The aim of this study was to evaluate the correlation between NT-proBNP and CCE and the potential usefulness of such variables during the weaning process from MV.

## Methods

Twenty-two long-term (>48 hours) mechanically ventilated patients capable of performing a weaning trial of spontaneous breathing (SBT) were enrolled in the study. Inclusion criteria were: age >18 years and equipment with a standard arterial catheter line. Exclusion criteria were: neuromuscular disease, tracheotomy, renal failure, and traumatic brain injury. During the weaning process, NTproBNP plasma levels, CCE, and standard hemodynamic and ventilatory data were collected 30 minutes before extubation (T1), 2 hours (T2) and 12 hours later (T3). After removal of tracheal tube, patients with a history of heart failure received continuous positive airway pressure (CPAP group). Patients with normal cardiac function were maintained with spontaneous breathing (SB group).

## Results

Sixty-six paired NT-proBNP and CCE values were obtained. Patients in the SB group and in the CPAP group were 10 and 12, respectively. In both groups there was a trend towards an increase in NT-proBNP values after extubation, an opposite trend was observed regarding CCE values (*P <*0.05). NT-proBNP levels showed an increase after extubation (T2, T3) compared with T1; conversely, CCE showed an inverse trend. Overall, a negative correlation was found between NT-proBNP and CCE values (*R *= -0.81, *P <*0.001). Significant inverse correlations were found between NT-proBNP and CCE at T1, T2, and T3 (*R *= -0.91, -0.75 and -0.73 respectively; *P <*0.001). The overall correlation between NT-proBNP and CCE was -0.74 in the SB group and -0.86 in the CPAP group. Standard hemodynamic and ventilatory data did not show significant changes during the study.

## Conclusion

NT-proBNP correlated well with CCE. The latter seems to be an additional attractive index of cardiovascular state that, in association with NT-proBNP changes, may provide information about cardiac function on a beat-by-beat basis during weaning process from MV.

